# Necroptosis in Intestinal Inflammation and Cancer: New Concepts and Therapeutic Perspectives

**DOI:** 10.3390/biom10101431

**Published:** 2020-10-10

**Authors:** Anna Negroni, Eleonora Colantoni, Salvatore Cucchiara, Laura Stronati

**Affiliations:** 1 Division of Health Protection Technologies, ENEA, 00123 Rome, Italy; 2 Maternal Infantile and Urological Sciences Department, Sapienza, University of Rome, 00161 Rome, Italy; eleonora.colantoni@uniroma1.it (E.C.); salvatore.cucchiara@unroma1.it (S.C.); 3 Department of Molecular Medicine, Sapienza University of Rome, 00161 Rome, Italy; laura.stronati@uniroma1.it

**Keywords:** programmed cell death, inflammation, cancer, intestinal diseases, inhibitors

## Abstract

Necroptosis is a caspases-independent programmed cell death displaying intermediate features between necrosis and apoptosis. Albeit some physiological roles during embryonic development such tissue homeostasis and innate immune response are documented, necroptosis is mainly considered a pro-inflammatory cell death. Key actors of necroptosis are the receptor-interacting-protein-kinases, RIPK1 and RIPK3, and their target, the mixed-lineage-kinase-domain-like protein, MLKL. The intestinal epithelium has one of the highest rates of cellular turnover in a process that is tightly regulated. Altered necroptosis at the intestinal epithelium leads to uncontrolled microbial translocation and deleterious inflammation. Indeed, necroptosis plays a role in many disease conditions and inhibiting necroptosis is currently considered a promising therapeutic strategy. In this review, we focus on the molecular mechanisms of necroptosis as well as its involvement in human diseases. We also discuss the present developing therapies that target necroptosis machinery.

## 1. Introduction

Cell death is crucial during the development and maintenance of tissue homeostasis in multicellular organisms. Until recently, apoptosis was considered the only molecularly controlled type of cell death, as opposed to necrosis, which was classically described as nonregulated accidental cell death. Several types of programmed necrotic cell death have been recently identified, which, although sharing common morphological features, such as cellular volume increase, organelles swelling, and plasma membrane disruption, however, respond to different triggers and follow distinct biochemical pathways [1]. Necroptosis is a programmed necrosis executed by the activation of death receptors including tumor necrosis factor receptor 1 (TNFR1), Fas receptor (FasR), TNF-related apoptosis-inducing ligand–death receptor 1 (TRAILR1), interferon receptor (IFNR) and also pattern-recognition receptors (PRRs) such as Toll-like receptors (TLRs) and RIG-like receptors (RLRs) [2,3,4]. 

The receptor-interacting protein kinase 1 (RIPK1) and 3 (RIPK3), as well as their target, the mixed lineage kinase domain-like protein (MLKL), are required to initiate necroptosis [5,6,7]. Necroptosis is associated with various human diseases including ischemic reperfusion injury, inflammatory, neurodegenerative, infectious, autoimmune diseases and cancer [8,9,10,11,12,13,14,15,16,17,18]. Recently, necroptosis has been also involved in mediating organ rejection in cardiac and renal allografts [8,12,19,20,21,22].

Despite the pathological connotation that characterized necroptosis at the beginning of its identification, emerging evidence points to its crucial role in physiological phenomena such as development, immunology and differentiation. For example, mutant mice with kinase-dead RIPK1 or RIPK3 and MLKL deficiency show no detrimental phenotype with regard to development and adult homeostasis [10]. Furthermore, several authors report that necroptosis is able to induce the innate immune response to viral infections, when the virus inhibits host apoptotic machinery and cells are committed to alternative cell death to limit viral replication or generate an immune response [23,24,25,26,27]. Necroptosis is also implicated in the regulation of antigen-activated T-cell proliferation and survival [28,29]. 

In this review, the molecular mechanisms of necroptosis as well as its role in the pathogenesis of gastrointestinal (GI) tract diseases and necroptosis-targeted therapies will be discussed. 

## 2. Necroptosis Signaling Pathway

**Figure 1:**
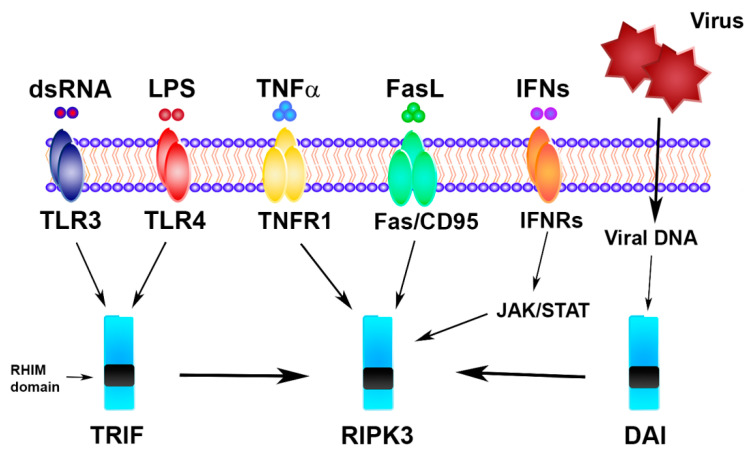
Necroptosis signaling triggers. TLR, Toll-like receptor; LPS, lipopolysaccharide; TNFα, tumor necrosis factor alpha; TNFR1, tumor necrosis factor receptor; FasL, Fas ligand; IFN, interferon; IFNR, interferon receptor; TRIF, Toll/interleukin-1 receptor (TIR) domain-containing interferon-β; DAI, DNA-dependent activator of IFN regulatory factors; RHIM, RIP-homotypic-interaction-motif domain; RIPK3, receptor-interacting protein kinase 3.

Necroptosis is a cellular response to environmental stress that can be caused by chemical and mechanical injury, inflammation, or infection. In addition to traditional triggers (Figure 1), recent evidence shows that necroptosis can be also induced by DNA damage, environmental stresses, such as the hypoxia and glucose level, and chemotherapeutic agents [30,31,32,33,34]. 

The mechanisms underlying the necroptosis pathway are mostly elucidated by a model of tumor necrosis factor alpha (TNFα)-induced cell death [8,9,35,36,37,38]. The binding of TNF to TNFR1 induces a conformational change in TNFR1 trimers, leading to the recruitment of a multiprotein platform named complex I, which includes RIPK1, TRADD (TNFR-associated death domain), cIAP1 (cellular inhibitor of apoptosis protein 1), cIAP2 and TRAF (TNFR-associated factor)2. The polyubiquitination of RIPK1 by cIAP1/2 causes the recruitment of complexes IKK (IKKα, IKKβ and NEMO) and TAK1 (TAK1, TAB1 and TAB2), leading to the activation of the transcription factor NF-kB and cell survival. The deubiquitination of RIPK1 by deubiquitinase cylindromatosis (CYLD) or the A20 ubiquitin-editing complex marks the transition from complex I to complex II and inhibits NF-κB activation [39]. TRADD and RIPK1 dissociate from TNFR1 recruiting Fad- associated protein with death domain (FADD) and pro-caspase-8 and leading to the formation of complex IIa, which results in the activation of caspase-8 cleavage followed by apoptosis [40].

The enrollment of RIPK3 causes the formation of complex IIb. Recent scientific advances have revealed the role of the cellular FLICE-inhibitory protein (cFLIP) as a critical switch to control cell survival and death in various tissues. cFLIP protein is evolutionarily conserved and expressed as functionally different isoforms, named long FLIP(L) and short FLIP(S) splice forms. A heteromeric complex between FLIP(L) and caspase-8 prevents the interaction of RIPK1 and RIPK3 in complex IIb, thereby preventing the induction of RIPK3-dependent necroptosis. In contrast, the binding of cFLIP(S) to FADD prevents the recruitment of caspase-8 and apoptosis [41,42,43]. Thus, the activity of caspase-8 is essential in determining the fate of a cell. A defect in the FADD-caspase-8 signaling pathway sensitizes cells towards necroptosis by promoting the assembly of the necrosome while preventing apoptosis. High levels of RIPK3 or caspase-8 inactivation, due to pharmaceutical or genetic intervention, promote the formation of the necrosome which is stabilized by the interaction and trans/autophosphorylation between RIPK1 and RIPK3. The RIP homotypic interaction motif (RHIM) domains on RIPK3 and RIPK1 are essential for their interaction. Interestingly, other RHIM-containing proteins, such as Toll/interleukin-1 receptor (TIR)-domain-containing adapter-inducing interferon-ß (TRIF) and the DNA-dependent activator of interferon-regulatory factor (DAI, also known as ZBP1), may interact and activate RIPK3, regardless of RIPK1, leading to necroptosis [35]. Afterwards, RIPK3 phosphorylates its substrate MLKL that oligomerizes and translocates to the plasma membrane to execute necroptosis [44,45,46,47,48].

**Figure 2:**
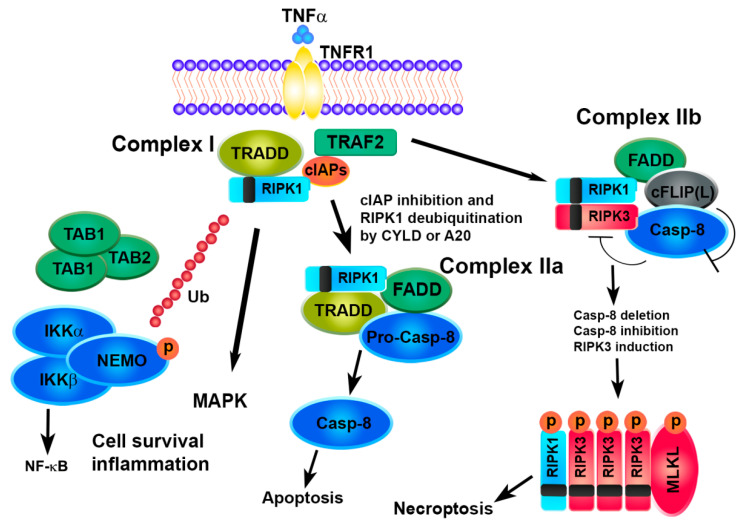
Survival or cell death pathways activated by TNFα/TNFR1. TNFα, tumor necrosis factor alpha; TNFR1, tumor necrosis factor receptor; TRADD, TNFR-associated death domain; TRAF2, TNFR-associated factor, cIAPs, cellular inhibitors of apoptosis protein, RIPK, receptor-interacting protein kinase, TAK1, transforming growth factor-activated kinase 1; TAB, TAK1-binding protein; IKK, inhibitor of NF-kB kinase; NEMO, NF-kB essential modulator; FADD, Fas-associated protein with death domain; CYLD, cylindromatosis lysine 63 deubiquitinase; cFLIP, cellular FLICE-like inhibitory protein; MLKL, mixed lineage kinase domain-like protein.

Recently, other effectors of necroptosis, downstream RIPK3, have been identified in addition to MLKL, such as the mitochondrial phosphoglicerate mutase 5 (PGAM5), [49] and Ca2+/calmodulin-dependent protein kinase (CAMK)-II [50]. PGAM5 exists as two splicing variants, the long form (PGAM5L) and the short form (PGAM5S). RIPK3, once activated, phosphorylates PGAM5L and then engages PGAM5S on the mitochondrial membrane. Activated PGAM5L/PGAM5S promote mitochondrial fission through the dephosphorylation of dynamin-related protein-1 (DRP1), thus leading to cellular necroptosis. CAMK-II is also phosphorylated by RIPK3 and once activated, regulates multiples ion channels opening with a consequent influx of extracellular ions and eventual plasma membrane damage, independently of MLKL [50] (Figure 2).

Necroptosis is finely controlled by several negative regulators in cells. For example, TBK1 and IKKε prevent TNF-induced cell death by RIPK1 phosphorylation [51,52]. Moreover, absent, transient and sustained levels of TAK1-mediated RIPK1 phosphorylation may represent distinct states in the TNFR1 signaling complex to dictate the activation of alternative cell death mechanisms [53]. The carboxyl terminus of Hsp70-interacting protein (CHIP, also known as STUB1) is a bona fide negative regulator of the RIPK1–RIPK3 necrosome formation leading to the desensitization of TNF-mediated necroptosis [54]. A20 (also known as TNFAIP3, TNF-induced protein 3) and ABIN-1 (also known as TNIP1, TNFAIP3-interacting protein 1), candidate susceptibility genes for several autoimmune or inflammatory diseases, synergistically restrict death by inhibiting TNF-induced caspase-8 activation and RIPK1 kinase activity by blocking both apoptosis and necroptosis [55].

Mice deficient in SET-domain-bifurcate-1 (SETDB1), a histone methyltransferase that mediates the trimethylation of histone H3 at lysine 9, develop necroptosis suggesting a suppressive role for the gene. Accordingly, SETDB1 is downregulated in patients with inflammatory bowel disease (IBD) [56].

## 3. Interplay between Necroptosis, Apoptosis, Pyroptosis and Autophagy

The interplay between necroptosis and other important cellular processes, such as apoptosis, autophagy or pyroptosis, and the identification of their converging points are crucial for developing novel therapeutic approaches in inflammatory diseases and cancer. Apoptosis occurs during the development as a homeostatic mechanism but also as a defense tool in immune reactions or in the presence of damaged cells [57].

In most situations, apoptosis is the default cell death modality, whereas necroptosis intervenes when key apoptotic mediators are blocked by pharmacological inhibition or genetic ablation or after a cellular stress such as energy ATP depletion or in certain viral infections [58,59].

Caspase-8 represents the molecular switch to control the balance between apoptosis and necroptosis [60,61]. Mice with the intestinal epithelial cell (IEC)-specific deletion of caspase-8 or its adapter FADD develop colitis and ileitis with a loss of Paneth cells and these effects are rescued by RIPK3 deficiency, underlying the importance of necroptosis in driving the pathology in both the small and large intestine [62,63].

Moreover, in mice with IECs, the specific deletion of caspase-8, the stimulation of TLR3 or TLR4 with microbial molecules (poly(I:C) and LPS) or after Salmonella Typhimurium infection causes an RIPK3-dependent epithelial necroptosis with more severe mortality and tissue damage [64,65]. Mice with IEC-specific FADD or caspase-8 deficiency develop colitis depending on MLKL-mediated epithelial cell necroptosis. Besides, caspase-8 and gasdermin-D (GSDMD), the effector of pyroptosis, a highly inflammatory form of regulated cell death which occurs most frequently upon infection with intracellular pathogens, are both required for the development of MLKL-independent ileitis in mice with epithelial FADD deficiency [66].

The execution of pyroptosis initiates the formation of a large supramolecular complex termed the inflammasome, and is regulated via caspases 1 and 11 which results in the activation of GSDMD and following pore formation in the plasma membrane with consequent citoplasmic swelling and the release of intracellular contents, including IL-1β and IL-18 [67].

Recent evidence reports that RIPK3 is able to activate the NLRP3 inflammasome-mediated pyroptosis to drive inflammation [68,69]. Moreover, MLKL protects from Salmonella infection promoting intestinal epithelial barrier function by inducing inflammasome activation in IECs. Besides, mice with MLKL ablation are more susceptible to Salmonella infection and present impaired caspase-1 and GSDMD cleavage with consequently decreased interleukin IL-18 release [70].

Transgenic (Tg) mice, wherein CFLARs, the gene encoding cFLIP, was integrated onto the X chromosome, die perinatally due to severe ileitis. The deletion of RIPK3 or MLKL prevented both necroptosis and apoptosis, and rescued the lethality of the CFLARs Tg mice [71].

A connection between necroptosis and autophagy was also discovered, albeit the molecular mechanisms remain poorly defined [72,73]. Autophagy regulates the degradation of cytoplasmic proteins and organelles within lysosomes, provides nutrients and energy under various stresses, including starvation, cellular and tissue remodeling, and cell death [74]. Recently, it has been shown that autophagy machinery can control programmed cell death, switching between apoptosis and necroptosis, by serving as a scaffold rather than as a degrading cellular material [75].

Moreover, the autophagy gene ATG16L1 is essential in the intestinal epithelium for preventing the loss of Paneth cells and a variant of ATG16L1 is associated with poor survival in allogeneic hematopoietic stem cell transplant recipients and Crohn’s disease (CD), one of the two forms of IBD. Intestinal organoids lacking ATG16L1 reproduce this loss in Paneth cells and display TNFα-mediated necroptosis, indicating that, in contrast to tumor cells in which autophagy promotes caspase-independent cell death, ATG16L1 maintains the intestinal barrier by inhibiting necroptosis in the epithelium [76]. It was also shown that ATG16L1 plays a role in coordinating the outcome of IL-22 signaling in the intestinal epithelium, leading to transient endoplasmic reticulum (ER) stress and the subsequent activation of type I interferon (IFN-I) signaling that amplifies epithelial TNF production and contributes to necroptotic cell death [77]. Besides, a critical function has recently emerged for the mammalian target of rapamycin (mTOR), an evolutionarily conserved protein kinase controlling the balance between cell growth and autophagy, in the regulation of RIPK3 expression and necroptosis in the gut epithelium. Epithelial mTOR hyperactivation, associated with the Western diet, dysbiosis, or the ablation of its repressor TSC1, inhibits RIPK3 degradation in autophagolysosome and promotes the necroptosis of epithelial cells, barrier disruption and chronic inflammation. Accordingly, hyperactive mTOR and aberrant necroptosis were intertwined in humans IBD [78].

Furthermore, the immunity-related GTPase family M protein (IRGM), that plays a role in innate immunity by regulating autophagy in response to several intracellular pathogens, regulates necroptosis and the release of damage-associated molecular patterns (DAMPs) inducing gastrointestinal inflammation. Interestingly, IRGM has been identified in genome-wide association studies as a genetic risk factor in CD. IRGM overexpression by phosphorylated eukaryotic translation initiation factor 2 (pEIF2A) plays a major role as a checkpoint allowing host cell survival by autophagy or host cell elimination by necroptosis [79].

Similarly, necroptosis may promote or repress autophagy, although the mechanisms are still unclear [80]. For example, necroptotic stimulation seems to reduce autophagic activity, as shown by the enlarged puncta of the autophagic substrate Sequestosome 1 (SQSTM1/p62) and its increased colocalization with microtubule-associated protein 1A/1B-light chain 3 (LC3), attenuating autophagic flux before the lysosome fusion step [81]. The activation of necroptosis in mouse dermal fibroblasts and HT-29 human colorectal cancer cells results in the accumulation of the autophagic marker, lipidated LC3B, in an MLKL-dependent manner. Unexpectedly, the necroptosis-induced increase in lipidated LC3B is due to the inhibition of the autophagic flux, not to the activation of autophagy [82].

## 4. Necroptosis and Inflammation

Molecular mechanisms of cell death have regulatory roles in inflammation and molecular changes associated with different forms of cell death may affect the course of the inflammation in different ways [83]. In recent decades, emerging knowledge on cell death and inflammation has enriched our understanding of the signaling pathways that mediate various programs of cell death and multiple types of inflammatory responses.

Necroptosis is typically considered a highly pro-inflammatory mode of cell death, due to the release of DAMPs, which can promote inflammation and activate an immune response, either alone (in the context of sterile injury) or in combination with pathogen-associated molecular patterns (PAMPs) [84,85,86]. Necroptosis promotes the activation of macrophages and dendritic cells which increases the levels of pro-inflammatory cytokines, including the IL-1 superfamily, thus triggering acute and chronic inflammatory diseases [5,36,84,85].

Recent studies reveal unexpected complexity in the regulation of cell death programs by RIPK1 and RIPK3 with the possibility that necroptosis is one mechanism by which these kinases promote inflammation [86]. For example, RIPK3 seems to control a separate, necrosis-independent pathway of inflammation by regulating NF-κB activation, dendritic cell (DC) biology, innate inflammatory-cytokine expression, and injury-induced tissue repair [87]. Several studies report that RIPK3 mediates the formation of NATCH, LRR and PYD domains-containing protein 3 (NLRP3) inflammasome and following the activation of caspase-1 and -11, through the interaction with different molecules, including caspase-8 and LPS [68,69,87,88,89,90]. It is unclear whether MLKL is involved in this process [87]. However, a recent paper showed that MLKL promoted intestinal epithelial barrier function by enhancing inflammasome activation [70].

In contrast to the traditional view of a pro-inflammatory mode of necroptosis, some authors suggest that it might have anti-inflammatory effects in certain settings, through curbing excessive TNF- or TLR-induced inflammatory cytokine production [36,37,91,92,93].

## 5. Necroptosis and Intestinal Diseases

### 5.1. Inflammatory Bowel Disease (IBD)

IBD is a chronic, debilitating intestinal disease with various clinical presentations whose main forms are ulcerative colitis (UC) and CD. The cause of IBD is still unknown, although it is believed to be a combination of multiple environmental factors along with genetic inheritance patterns, all leading to an excessive and abnormal immune response against commensal gut flora [94].

The first demonstration of the involvement of necroptosis in the pathogenesis of IBD derives from evidence showing that the genetic ablation of either caspsae-8 or Fadd in IECs is sufficient to induce necroptosis and spontaneous ileitis and/or colitis [62,63]. Later, RIPK3 overactivation is observed in the inflamed tissues of pediatric and adult IBD patients [62,95]. A recent paper confirmed the positive correlation between the upregulation of necroptosis and the severity of disease in IBD patients [96].

Moreover, subjects carrying a homozygous loss-of-function mutations in RIPK1 show early-onset IBD [97].

Recently, our group showed that RIPK3-driven necroptosis seriously affects intestinal inflammation by increasing pMLKL, activating different cytokines and alarmins, and altering epithelial permeability. The inhibition of necroptosis causes a significant decrease in all these effects [98]. Accordingly, the use of the RIPK1 inhibitor necrostatin-1 (nec-1) reduces the intestinal inflammation and colitis-associated tumor growth in mice with dextran sulfate sodium ((DSS)-induced colitis [99]) while MLKL deficiency inhibits colitis by preventing inflammatory cytokines production and MAPK signaling activation [100]. MLKL^-/-^ mice are highly susceptible to colitis and colitis-associated tumorigenesis, associated with massive leukocyte infiltration and increased inflammatory responses, suggesting a protective role of the necroptosis effector [91]. Furthermore, the necroptosis inhibitor necrosulfonamide (NSA) represses necroptosis in intestinal epithelial cells in vitro suggesting a potential beneficial effect against IBD [101].

Lately, the expression of a secreted form of interferon lambda (IFNλ) in mice resulted in a loss of Paneth cells from intestinal tissues, via STAT1 and MLKL, controlled by caspase-8 [102].

The inhibition of HtrA2 by the serine protease UCF-101 alleviates DSS-induced colitis by preventing the necroptosis of intestinal epithelial cells [103]. Furthermore, an unexpected connection between necroptosis and the members of the disintegrin and metalloproteinase (ADAM) protease family is also described [104]. Indeed, murine embryonic fibroblasts derived from *ADAM17* ^ex/ex^ mice fail to show the phosphorylation of *MLKL* and *RIPK3* after TNF-induced necroptosis, suggesting a role of *ADAM17* in this process [105].

In a recent study, Lee and Coll. [106] observe that the expression of proinflammatory cytokines in the peripheral blood mononuclear cells of UC patients and in mice with a DSS-induced colitis is markedly decreased by the administration of the RIPK3 inhibitor GSK872.

The circadian rhythm disruption (CRD) is known to be a risk factor for IBD. The genetic ablation of circadian clock function or environmental CRD in mice increases susceptibility to severe intestinal inflammation and epithelial dysregulation, accompanied by excessive necroptotic cell death and a reduced number of secretory epithelial cells, thus suggesting that CRD increases intestinal necroptosis, rendering the gut epithelium more susceptible to inflammatory processes [107].

### 5.2. Necrotizing Enterocolitis (NEC)

Necrotizing enterocolitis (NEC) is a rare but devastating GI disease that predominately affects preterm neonates [108]. The pathogenesis of NEC is incompletely understood and appears to be multifactorial. From a mechanistic viewpoint, it has been shown that the bacterial activation of the innate immune receptor TLR4 in the intestinal epithelium leads to barrier injury and the inflammatory microenvironment is required for the development of NEC. Indeed, the expression of TLR4 in the intestinal epithelium is higher in the premature vs. full-term intestine and mice lacking TLR4 in the intestinal epithelium are protected from NEC [109].

Recently, necroptosis has been shown to be activated in the intestinal epithelium upon TLR4 signaling and is required for NEC development [110]. The imbalance of IEC cell death, resulting in increased intestinal permeability and barrier dysfunction, leads to several acute and chronic intestinal diseases, including NEC [111].

Recently, a study shows that miR-141-3p protects IECs from LPS damage by suppressing RIPK1-mediated inflammation and necroptosis, providing an alternative perspective to explore the pathogenesis of NEC [112].

### 5.3. Bacterial Intestinal Infections

Infections with bacterial pathogens often results in the initiation of programmed cell death, including necroptosis, as part of the host innate immune defense, or as a bacterial virulence strategy [113].

The sexually transmitted pathogen Chlamydia trachomatis (CT) is able to replicate and survive in human IECs where induces a cytotoxic effect by various mechanisms, such as oxidative stress, apoptosis and necroptosis. Intracellular bacterial infections release DNA, sensed by DAI or TLR9, or ligands that can activate TRIF and IFN signaling that induce necroptosis, based upon the established interactions of the RHIM-containing proteins, RIPK1, RIPK3, TRIF, and DAI. Indeed, RIPK1 inhibition significantly reduces the mortality of infected cells. Authors speculate that epithelial cells, through the activation of necroptosis, can prevent the completion of pathogen replication cycles, thus blocking the progression of CT infection [114].

Moreover, Salmonella outer protein B (SopB) has a role in modulating necroptosis to facilitate the bacteria escape the epithelial cell and spread to systemic sites through a Salmonella-induced colitis model [115], while the caspase-8 knockout mice infected with Salmonella typhimurium show more severe mucosal injury and intestinal epithelial cell death as compared to wild-type mice [64].

Listeria monocytogenes, a bacterial foodborne pathogen, efficiently spreads and causes systemic infection in RIPK3-deficient mice while almost no dissemination is observed in wild-type mice. Intriguingly, MLKL is shown to directly bind to Listeria and inhibits their replication in the cytosol, suggesting a novel functional role of the RIPK3–MLKL pathway in nonimmune cell-derived host defense against Listeria invasion [116]. Clostridium perfringens uses its large arsenal of protein toxins to produce histotoxic, neurologic and intestinal infections in humans and animals, resulting in the activation of intracellular pathways with a variety of effects, commonly including cell death, such as apoptosis, necrosis and/or necroptosis [117]. MLKL^-/-^ mice are more susceptible to Salmonella infection compared to their wild-type counterparts, with higher mortality rates, increased body weight loss, exacerbated intestinal inflammation, more bacterial colonization, and severe epithelial barrier disruption [70]. 

Alterations of the necroptosis key genes in intestinal human diseases are listed below (Table 1).

## 6. Necroptosis and GI cancer

Recent research attributes to necroptosis both a tumor-suppressing and tumor-promoting activity depending on the type, stage and localization of the tumor. The tumor-suppressing activity of necroptosis has been shown in many types of cancer in which the expression of RIPK3 [118,119] or MLKL [120] is silenced by methylation [18,34,121] or altered by polymorphisms in coding genes [118,122], suggesting that cancer cells evading apoptosis may also escape from necroptosis [13,18]. Hence, RIPK3 expression is downregulated in many cancer cell lines [18,123] as well as in several human cancers, including breast cancer [18], colorectal cancer (CRC) [17], acute myeloid leukemia (AML) [124] and melanoma [123]. The induction of necroptosis may therefore also represent an alternative strategy for killing cancer cells [125,126].

The tumor-suppressing activity of RIPK3 has been documented in CRC, and its overexpression significantly reduces the proliferation, migration and invasion of cancer cells in vitro. Accordingly, low RIPK3 expression is associated with poor clinical outcomes in human CRC [17]. Moreover, RIPK3 knockout mice have a higher risk of developing colitis-associated CRC by producing an increased amount of pro-inflammatory or tumor-promoting factors, such as the transcription factor STAT3 [92]. Although the signaling events that induce different forms of programmed cell death are well defined, the subsequent immune responses to dying cells in the context of cancer remain relatively unexplored. A role for RIPK1/RIPK3 activation has been proposed as a beneficial proximal target in the initiation of tumor immunity, so that maximizing the immunogenicity of dying cells within the tumor microenvironment through the specific activation of necroptosis may represent an advantageous treatment approach [127].

Remarkably, the release of cell content, including DAMPs and cytokines/chemokines, characterizing necroptosis, underlies the immunogenic nature of necroptotic cancer cells and their ability to induce efficient antitumor immunity [128]. As well, decreased RIPK3 in CRC boosts tumorigenesis via the accumulation and immunosuppressive activity of myeloid-derived suppressor cells (MDSCs). Indeed, decreased RIPK3 in MDSCs and CRC elicits NF-κB-transcribed COX-2, which catalyzes the synthesis of prostaglandin E_2_ (PGE_2_). In turn, PGE_2_ exacerbates the immunosuppressive activity of MDSCs and accelerates tumor growth [129].

Otherwise, some authors suggest that inflammation underlying necroptosis may promote tumor development by providing genomic instability, angiogenesis, cell proliferation, and accelerating metastasis [130]. A higher phosphorylation level of MLKL has been correlated with a poorer prognosis and shorter survival in human patients with colon and esophageal cancer and the use of the MLKL inhibitor, NSA, was shown to significantly delay the tumor growth, highlighting the role of necroptosis in tumor promotion [131]. However, the role of necroptosis in cancer is complex and further precise characterization of necroptosis will be critical for the validation of the significance of necroptosis in different types of cancer.

In vivo and ex vivo models of necroptosis are listed below in Table 2.

## 7. Therapeutic Perspectives

In recent decades, promising therapies targeting different signaling pathways have emerged. Emerging evidence has shown that bypassing apoptosis-induced cell death is a mechanism of multidrug resistance; hence, the modulation of necroptosis seems to be a promising approach to overcoming apoptotic drug resistance [132,133].

Here, we summarize a panel of compounds that targets necroptosis, some of which were successfully experimented with in preclinical and clinical trials [8,19,131].

Necrostatins are tryptophan-based compounds that inhibit the kinase activity of RIPK1 [134,135]. Nec-1 significantly decreases intestinal inflammation in vitro and in cultured intestinal explants from IBD [102] and reduces colitis-associated tumorigenesis in mice [103]. Nec-1 and the stable modified form, nec-1s, prevent necroptosis, although with moderate potency and poor pharmacokinetic properties [134,135].

Furthermore, RIPK1 is the cellular target of a new series of type III kinase inhibitors, of which the highly potent and selective compound is the *N*-benzyl-*N*-hydroxy-2,2-dimethylbutanamide (RIPA-56), that shows an impressive and efficient protection of mice from TNFα-induced mortality and multiorgan damage in the systemic Inflammatory Response Syndrome (SIRS) disease [136].

Other RIPK1 inhibitors have been developed by GlaxoSmithKline, among which GSK2982772 is currently in phase 2a clinical studies for psoriasis, rheumatoid arthritis, and ulcerative colitis [137,138] and GSK3145095 has terminated the phase I clinical trials for solid tumors [8].

Small molecule RIP3 inhibitors, GSK’840, GSK’843 and GSK’872, have been identified targeting RIPK3 kinase and inhibiting necroptosis, although their therapeutic value is undermined by a surprising, concentration-dependent induction of apoptosis [139,140].

B-Raf(V600E) inhibitors, such as Dabrafenib, are an important anticancer drug class for metastatic melanoma therapy, that seem to inhibit the RIPK3 enzymatic activity in vitro, through their ATP-competitive binding to the enzyme [141]. The pan-RAF inhibitor LY3009120 is a necroptosis inhibitor as well and may serve as a potential therapeutic drug for colitis [142].

Recently, a novel class of RIPK1/RIPK3 dual inhibitors, which effectively block necroptosis in both human and murine cells, have been identified [143]. For example, two receptor tyrosine kinase inhibitors, Pazopanib, suggested for treating advanced renal cell carcinoma and soft tissue sarcoma, and Ponatinib, suggested for treating chronic myeloid leukemia and Philadelphia chromosome-positive acute lymphoblastic leukemia, are found to inhibit necroptosis induced by various cell death receptor ligands in human cells, while not protecting from apoptosis. [144]. Both inhibitors target the ATP pocket, but Ponatinib also binds to an allosteric site behind the ATP pocket, named back pocket with different affinities. Consistent with their different allosteric properties, Ponatinib targets RIPK1, RIPK3 and MLKL, while Pazopanib preferentially targets RIPK1. The two compounds successfully block necroptosis, but their clinical application is not promising due to their cardiotoxicity [144].

MLKL inhibitors provide a recent powerful tool to study the biological function of the protein and demonstrate that MLKL is a druggable target. NSA binds only to the human variant of MLKL, so it is not valuable in preclinical studies (101). A novel class of MLKL inhibitors with single nanomolar potency (compound 15 is also named as TC13172) that covalently binds to Cysteine-86 (Cys-86) of the kinase, is under investigation [145].

Inhibitors of heat shock protein 90 (HSP90), a molecular chaperone controlling the RIPK3 and MLKL stability and function, such as kongensin A, 17AAG, IPI-504 and Alvespimycin (17-D MAG), both in clinical trials in cancer patients, may indirectly impair necroptosis [146].

Various microRNAs (miRNAs), endogenous non-coding RNAs involved in gene expression control necroptosis. MiR-155 prevents necroptosis in human cardiomyocyte progenitor cells by directly targeting RIPK1 [147]. CLYD, the key deubiquitinating enzyme in the apoptosis/necroptosis pathway, is directly targeted by miR-181b-1 and miR-19 in cancer cells, causing the hyperactivation of the NF-κB signaling pathway, increased inflammation and tumor progression [148,149].

**Figure 3:**
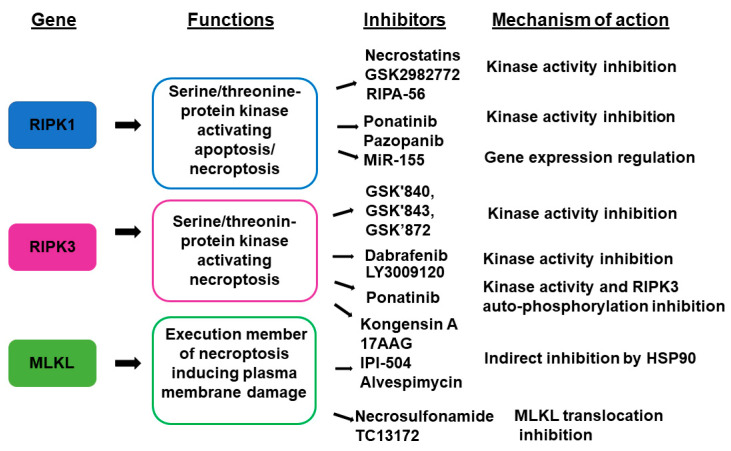
Key necroptotic genes and their inhibitors.

Several natural compounds are modulators of necroptosis. Hesperetin, a flavonoid from citrus fruits inactivates RIPK3/MLKL signaling improving the DSS-induced colitis in mice [150]. Furthermore, flaxseed oil, rich in n-3 polyunsaturated fatty acid (PUFA) α-linolenic acid (ALA), attenuates LPS-induced intestinal injury by impairing necroptosis and TLR4/NOD signaling in a piglet model. [151]. Recently, celastrol, a triterpene from the root bark of the Chinese medicinal plant, Tripterygium wilfordii, has been shown to inhibit necroptosis in a DSS mice model [152] (Figure 3).

Targeting necroptosis is a therapeutic strategy in apoptosis-defective cancer cells.

Necroptosis cell death is identified as an important effector mechanism of 5-fluorouracil (5-FU)-mediated anti-tumoral activity [153]. Moreover, RIPK3, highly expressed in mouse models of CRC and in a subset of human CRC cell lines, seems to be the deciding factor of cancer cell susceptibility, second to mitochondria-derived activator of caspase (SMAC) mimetic-induced necroptosis [154]. The 3-Bromopyruvate (3BP) can induce colon cancer cell death by necroptosis and apoptosis at the same time and concentration in the SW480 and HT29 cell lines [155]. More recently, resibufogenin, a member of the bufadienolide family, is proven to suppress the growth and metastasis of CRC by inducing necroptosis in vivo through the upregulation of RIPK3 and the phosphorylation of MLKL at Ser358 [156].

The topoisomerase inhibitor SN38, an active metabolite of irinotecan, causes cytotoxicity through the TNF/TNFR signaling pathway in a panel of colon cancer cells and promotes the progression of necroptosis, [123], suggesting its potential use in the treatment of CRC [157].

A recent paper proposes a generic antitumor therapy based on the intratumor delivery of mRNA that codes for the necroptosis effector MLKL, showing that this intervention stalls primary tumor growth and protects against distal and disseminated tumor formation in syngeneic mouse melanoma and colon carcinoma models [158].

Necroptosis is also proposed to increase the sensitivity to radio- and chemiotherapy through the activation of the RIP1/RIP3/MLKL/JNK/IL-8 pathway. If confirmed by further studies, this view might open new therapeutic perspectives, strengthening the role of necroptosis in cancer therapy [159].

## 8. Conclusions

Necroptosis is now recognized as a major form of regulated cell death. It is mediated by RIPK1/RIPK3 activation, followed by MLKL phosphorylation and membrane disruption. Necroptosis is becoming increasingly attractive as a field of study given its implications in the pathogenesis of inflammatory diseases and cancer, including those of the GI tract. Targeting RIPK3 and RIPK1 may help to overcome therapeutic hurdles in the treatment of these complex disorders. Although the necroptosis field has developed rapidly in recent years, it also faces many challenges. For example, how cells select survival, apoptosis, necrosis and necroptosis according to different physiological and pathological conditions, the regulation of RIPK family activity and its substrates or molecular mechanisms of MLKL. It is also necessary to unveil the molecular mechanism through which the necroptosis process regulates the phenotype of immune cells in the gut.

Through further investigation of the molecular mechanism of necroptosis, we will be more aware of cell death and develop new ideas for the exploration of the pathogenesis of various human diseases.

## Figures and Tables

**Table 1:** Alterations of necroptotic genes in human intestinal diseases. IBD, inflammatory bowel disease; UC, ulcerative colitis; CD, Crohn’s disease; NEC, necrotizing enterocolitis; pMLKL, phospho-MLKL; pRIPK3, phospho-RIPK3; PBMC, peripheral blood mononuclear cell; nec-1, necrostatin-1.

Gene Alterations	Human Model	Effects	Ref.
RIPK3, MLKL, pMLKL upregulation	UC inflamed biopsies		[106]
RIPK3 inhibition	PBMC from UC patients treated with GSK 872	Necroptosis and proinflammatory cytokines reduction	[106]
pRIPK3, pRIPK1, pMLKL upregulation	inflamed biopsies	Increased necroptotic cell death	[96]
RIPK3 protein expression	Colon cancer patients with metastatic stage	High expression of RIPK3 associated with lower risk of disease progression	[118]
RIPK3, MLKL protein upregulation	CD inflamed biopsies and serum	Paneth cells necroptosis correlates with high level of INFλ in serum	[102]
RIPK3, MLKL protein upregulation	NEC surgical specimen	Increased necroptosis	[110]
loss-of-function mutations in RIPK1	IBD patients	Predisposition to viral, bacterial and fungal infections, early-onset IBD, arthritis	[97]
homozygous loss-of-function mutations in RIPK1	Skin fibroblasts from IBD patients stimulated with TNFa or poly(I:C)	Increased necroptosis	[97]
RIPK3 and pMLKL protein upregulation	Inflamed biopsies from IBD pediatric patients	Increased epithelial permeability, cytokine and alarmin expression	[98]
RIPK1 inhibition	Colonic tissue culture from pediatric CD treated with nec-1	Proinflammatory cytokines reduction	[98]
RIPK3, MLKL protein upregulation	Pediatric IBD inflamed biopsies		[95]
RIPK3 upregulation	CD inflamed biopsies	Loss of Paneth cells	[62]

**Table 2:** In vivo and ex vivo studies reporting necroptotic genes alterations. IEC, intestinal epithelial cells; DSS, dextran sulfate sodium, IFN, interferon; AOM, azoxymethane; i.p., intraperitoneal; NEC, necrotizing enterocolitis; CAC, colitis-associated cancer; SMAC, second mitochondria-derived activator of caspase; Nec-1, necrostatin-1; GSK547 (RIPK1 inhibitor); MNV, murine norovirus; LPS, lipopolysaccharide; poly (I:C), polynosine–polycytidylic acid (I:C); CRC, colorectal cancer; NSA, necrosulfonamide; zVAD-FMK, carbobenzoxy-valyl-alanyl-aspartyl-(O-methyl)- fluoromethylketone (pan-caspase inhibitor); IL, interleukin; BafA, bafalomycin A.

Gene Alteration	Model	Effects	Ref.
**In Vivo:**			
FADD^∆IEC^/RIP3-/-or MLKL-/-Casp8^IEC^/RIP3-/- or MLKL-/-		Prevention of colitis, cell death and inflammation	[66]
Setdb1^∆IEC/^Ripk3-/- or Mlkl-/-		Inhibition of stem cell death	[56]
MLKL^IEC^	High-protein diet and DSS-induced colitis.	Rescue of IEC death and intestinal inflammation	[78]
IEC Casp8 ^C362S/C362S^ Mlkl−/−		Severe intestinal inflammation	[61]
Ripk3−/− or Mlkl−/− Casp8^IEC^	IFNλ injection	Rescue of INF induced necroptosis in Paneth cells	[102]
cFLIPs Tg mice RIPK3-/- or MLKL-/- or RIPK1^DN/DN^		Partially rescue of lethality, epithelial cell death and villous destruction	[71]
Mlkl-/-	AOM i.p. injection + DSS-induced colitis	Susceptibility to colitis and colitis-associated tumorigenesis	[91]
Mlkl-/-	DSS-induced colitis	Inhibition of intestinal inflammation independent from microbiota	[100]
Mlkl-/-	DSS-induced colitis	Prevention of body weight loss and mortality	[103]
Ripk3−/− or Mlkl−/−	NEC	Reduced IEC death and mucosal inflammation	[110]
Casp8^∆IEC^Ripk3−/− or Mlkl−/−	*Salmonella*-induced gastroenteritis	Rescue of IEC death, body weight loss and mucosal destruction	[64]
Ripk3-/-	AOM + DSS-induced CAC	Promotion of colorectal carcinogenesis and infiltration of myeloid-derived suppressor cells	[129]
Mlkl-/-	*Salmonella*-induced colitis	Rescue of barrier integrity	[70]
ATG16L1^IEC^	MNV + DSS-induced colitis + GSK547	Rescue of clinical score	[76]
Casp8^∆IEC^Ripk3-/-	LPS or poly (I:C) i.p. injection	Rescue of IEC death and destruction of crypt-villus architecture	[65]
Ripk3-/-	Induced CRC	Higher susceptibility to CRC	[92]
Ripk3-/-_	DSS-induced colitis+LPS	Higher susceptibility to colitis	[86]
Chip-/-/Ripk3-/-		Reduction of cell death induced by Chip deletion in the small intestine	[54]
Ripk3-/-	DSS-induced colitis	RIPK3 protective against colitis	[87]
Casp8^IEC^	Spontaneous ileitis	Increased cell death of Paneth cells	[62]
Fadd^IEC^	Spontaneous colitis	IEC cell death, loss of Paneth cells, enteritis and severe colitis.	[63]
**Ex Vivo:**			
IEC TSC1^IEC^ RIP3+/- organoid	Z-VAD-FMK + poly I:C/TNFα/IFNβ	Cell death attenuation	[78]
MLKL^IEC^ organoid	IL-22 + BafA	Reduced cell death	[77]
ATG16L1^IEC^ organoid	Z-VAD-FMK + Nec-1	Inhibition of cell death	[76]
Casp8^IEC^ organoid	Poly I:C + Nec-1	Inhibition of cell death	[65]
Casp8^IEC^ organoid	TNFα + Nec-1	Inhibition of cell death	[62]
